# Expression of the "stem cell marker" CD133 in pancreas and pancreatic ductal adenocarcinomas

**DOI:** 10.1186/1471-2407-8-48

**Published:** 2008-02-08

**Authors:** Heike Immervoll, Dag Hoem, Per Øystein Sakariassen, Ole Johnny Steffensen, Anders Molven

**Affiliations:** 1 Section for Pathology, the Gade Institute, University of Bergen, Bergen, Norway; 2 Department of Pathology, Haukeland University Hospital, Bergen, Norway; 3 Department of Surgery, Haukeland University Hospital, Bergen, Norway; 4 Department of Biomedicine, University of Bergen, Bergen, Norway; 5 Department of Pathology, Ålesund Hospital, Ålesund, Norway

## Abstract

**Background:**

It has been suggested that a small population of cells with unique self-renewal properties and malignant potential exists in solid tumors. Such "cancer stem cells" have been isolated by flow cytometry, followed by xenograft studies of their tumor-initiating properties. A frequently used sorting marker in these experiments is the cell surface protein CD133 (prominin-1). The aim of this work was to examine the distribution of CD133 in pancreatic exocrine cancer.

**Methods:**

Fifty-one cases of pancreatic ductal adenocarcinomas were clinically and histopathologically evaluated, and immunohistochemically investigated for expression of CD133, cytokeratin 19 and chromogranin A. The results were interpreted on the background of CD133 expression in normal pancreas and other normal and malignant human tissues.

**Results:**

CD133 positivity could not be related to a specific embryonic layer of organ origin and was seen mainly at the apical/endoluminal surface of non-squamous, glandular epithelia and of malignant cells in ductal arrangement. Cytoplasmic CD133 staining was observed in some non-epithelial malignancies. In the pancreas, we found CD133 expressed on the apical membrane of ductal cells. In a small subset of ductal cells and in cells in centroacinar position, we also observed expression in the cytoplasm. Pancreatic ductal adenocarcinomas showed a varying degree of apical cell surface CD133 expression, and cytoplasmic staining in a few tumor cells was noted. There was no correlation between the level of CD133 expression and patient survival.

**Conclusion:**

Neither in the pancreas nor in the other investigated organs can CD133 membrane expression alone be a criterion for "stemness". However, there was an interesting difference in subcellular localization with a minor cell population in normal and malignant pancreatic tissue showing cytoplasmic expression. Moreover, since CD133 was expressed in shed ductal cells of pancreatic tumors and was found on the surface of tumor cells in vessels, this molecule may have a potential as clinical marker in patients suffering from pancreatic cancer.

## Background

Organ stem cells are slow-cycling cells with the capacity of unlimited self-renewal, asymmetric cell division and differentiation into mature cell types. The concept of stem cells as a definite cell population in a supportive microenvironment (niche) is now widely accepted, and regarded as the source for tissue renewal [1]. Recent studies suggest that a small population of cells with unique self-renewal properties and malignant potential exists in leukemia [2,3] and in at least some solid tumors [4-6]. However, these "cancer stem cells" are not a well-defined entity and should still be considered a hypothesis for further exploration. Whether a tumor derives from transformed organ stem cells or whether the cancer stem cells have acquired their self-renewal capacity during tumor development is an open question. If the model were correct, the slow-cycling cancer stem cells would escape current treatments designed to kill cycling cells and should be the preferred target population for new therapies [7-10].

In the adult pancreas, a clear identification of stem cells has so far not been achieved (reviewed in [11-13]). Due to the microanatomy of this organ with exocrine and endocrine components intimately connected and its embryonic origin from two separate evaginations of the primitive gut epithelium, potential stem cell markers are not straightforward to predict from knowledge about pancreatic development. Stem cells of the pancreas therefore remain to be unequivocally identified, even though the research activity is high because of the urgent need of a resource of beta-cells for transplantation in patients suffering from insulin-dependent diabetes mellitus. Candidates for pancreatic stem cells have been suggested to reside both in the ductal epithelium, in the islets and among the acinar cells [14-16]. Further controversies arise from studies that have reported self-renewal of beta-cells [17,18], transdifferentiation of acinar cells [19] and cellular contributions from the bone marrow [20]. Li and co-workers recently described a subpopulation of tumor cells from pancreatic cancer tissue with increased tumorigenic potential in mice [21]. However, studies of potential cancer stem cells from human solid tumors have some immanent problems due to the necessity of tissue processing before cell sorting and of transfer to another species for functional testing.

Expression of the surface protein CD133 (also known as AC133 and prominin-1) is one criterion which has been used in the identification of putative cancer stem cells from solid tumors (brain: [5,6], lung: [22], skin melanoma: [23], prostate: [24], kidney: [25], colon: [26,27], liver: [28]). CD133 is expressed in a variety of cell lines, developing epithelia and differentiated cells in mammals and metazoans (reviewed in [29,30]). It was originally regarded as a marker for stem and progenitor cells of the hematopoietic system [31,32]. The human CD133 protein is encoded by the *PROM1 *gene on chromosome 4p15 and codes for a five-transmembrane glycoprotein [33,34]. Structurally, it consists of an N-terminus on the extracellular side, two short intracellular loops, two large extra cellular loops containing eight N-linked glycosylation sites, and an intracellular C-terminus [33,35]. CD133 localizes to plasma membrane protrusions at the apical surface of cells, reflecting a polarized cell structure [35-39]. The function of the protein is unknown. Examination of a family with autosomal recessive retinal degeneration revealed a *PROM1 *mutation resulting in a truncated form of CD133, which was not transported to the cell surface [40].

CD133 mRNA has been shown to be upregulated in shed ductal cells from pancreatic cancer patients [41] and in two pancreatic cancer cell lines [42]. The protein expression pattern of CD133 has not been thoroughly examined in the normal pancreas or pancreatic malignancies. By using immunohistochemical methods, we sought to perform a characterization of the tissue distribution of this protein in formalin-fixed, paraffin-embedded human tissue, with particular emphasis on the pancreas. We found that the amount of cancer cells expressing CD133 on their surface is far too high to be restricted to a cancer stem cell population. However, we noted a subpopulation with predominantly cytoplasmic positivity, which stained similarly to some single cells in the normal duct epithelium and to cells in centroacinar position. These are suggested sites for stem cells of the adult pancreas [15,16] and for the origin of pancreatic intraepithelial neoplasia [43,44], a precursor lesion of infiltrative ductal adenocarcinomas.

## Methods

### Tissue specimens

Tissue specimens from normal human organs and tumors, including 51 pancreatic adenocarcinomas resected in the period 1997–2004, were retrieved from the archives of the Department of Pathology, the Gade Institute, Haukeland University Hospital. A pathologist (H.I.) reviewed the slides to ensure that the cases were consistent with pancreatic ductal adenocarcinoma according to the WHO classification [45]. Clinical records and radiological reports were reviewed by a surgeon (D.H.). The pancreatic tumor samples are an extended series of that described by Immervoll et al. [46]. The study was approved by the Regional Ethics Committee and performed according to the Helsinki Declaration.

### Tissue micro-arrays

For construction of tissue micro-arrays (TMA) of formalin-fixed, paraffin-embedded pancreatic tumors, areas containing cancerous and normal tissue were identified in hematoxylin-eosin (H&E) stained slides. The TMAs were manually constructed using a commercial tissue micro-arrayer (Beecher Instruments, Silver Spring, MD). The TMA blocks consisted of four columns of cylindrical tumor tissue fragments and one column of a cylindrical fragment of tumor-free pancreatic tissue for each case. The cylindrical fragments were 1 mm in diameter and two punch-outs were put on top of each other to obtain a height of 4 mm. Tissue fragments were melted into the acceptor paraffin block by heating the block for 30 minutes at 39°C.

### Immunohistochemistry

The primary antibodies employed are listed in Table 1. Immunohistochemical staining was done on 3–4 μm-sections from formalin-fixed, paraffin-embedded tissues, placed on coated glass slides, and dried at 60–70°C for 30 min. For CD133, retrieval methods, buffer solutions, incubation time and concentrations of the primary antibodies were varied to find the optimal procedure. As antibody detection method, two polymer-based systems and one streptavidin-biotin system were tested (EnVision and LSAB, both from Dako, Glostrup, Denmark; MACH3 Polymer Detection Kit from Biocare Medical, Concord, CA). For double-labeling with cytokeratin 19 (CK19) or chromogranin A, standard methods employed in routine diagnostic service were used with antibody concentrations adjusted (Table 1).

**Table 1:** Primary antibodies used for immunohistochemistry

Antigen	Antibody subtype	Dilution	Product no.	Manufacturer
CD133/1 (clone AC133)	Mouse monoclonal	1:25	130-090-422	Miltenyi Biotec, Bergisch Gladbach, Germany
CD133/2 (clone AC141)	Mouse monoclonal	1:25	130-090-423	Miltenyi Biotec, Bergisch Gladbach, Germany
CD133 (K-18)	Goat polyclonal	1:50	sc-23797	Santa Cruz Biotechnology, Santa Cruz, CA
Cytokeratin 19	Mouse monoclonal	1:150	M0888	Dako, Glostrup, Denmark
Chromogranin A	Rabbit polyclonal	1:3000	A0430	Dako, Glostrup, Denmark

The CD133 detection method that we judged to be most sensitive and specific was the following: Antigen retrieval was performed by incubation in a pressurized heating chamber (Pascal, Dako) at 120°C for 1 min in Tris-EDTA buffer (pH 9). The slides were then cooled in running tap water and incubated with primary antibody (clone AC133; Miltenyi, Bergisch Gladbach, Germany) diluted 1:25 in an antibody diluent with reduced salt concentration (25 mM Tris, 75 mM NaCl, 1% BSA, 0.01% methiolate, 0.05% Tween 20; pH 7.4) for 60 min. This and all subsequent steps were carried out at room temperature. Next, blocking for unspecific peroxidase activity was done by 3% H_2_0_2 _treatment for 5 min. Primary antibody detection was performed, in accordance with the manufacturer's instructions, with the MACH3 mouse probe (Biocare Medical) for 20 min, followed by MACH3 HRP polymer (Biocare Medical) for 20 min, and the signal was developed with diamino-benzidine DAB+ (Dako) for 5 minutes. Between each step, there were two washing steps for 1 min each on a rocking platform in washing buffer (50 mM Tris, 150 mM NaCl, 0.05% Tween 20; pH 7.5). Finally, the slides were counter-stained with hematoxylin for 1 min, dehydrated in alcohol solutions and xylene, and mounted in Entellan (Merck, Darmstadt, Germany).

### Assessment of immunohistochemical staining

The quality of staining was judged in control material from different organs, according to the data in the literature about gene/protein expression of CD133 in various tissue types [33,35,37,47]. TMA slides containing pancreatic ductal adenocarcinomas were scored independently by two of the authors (H.I., A.M.) as negative (0), weakly positive (1) or strongly positive (2) for CD133 expression. Cases with different scoring were discussed to reach a consensus. As validation for TMA interpretation, whole sections from the border between adenocarcinoma and nearby normal pancreatic tissue were made from ten of the cases included in the TMA block, and treated and evaluated in the same way as the TMA slides. Parallels were stained with H&E for control of the tissue quality. The whole sections were also screened at 1000× magnification, looking for features such as nuclear/cytoplasmic staining, expression in vessels etc.

### Western blot

Snap-frozen tissue from patient biopsies was crushed in liquid nitrogen, dissolved in lysis buffer (20 mM MOPS, 5 mM EDTA, 2 mM EGTA, 30 mM NaF, 0.5% Triton X, 40 mM b-glycerophosphate, 20 mM Na-pyrophosphate, 1 mM Na-orthovanadate, 3 mM benzamidine, 5 μM Pepstatin, 10 μM Leupeptin, 1 mM PMSF; pH 7.2), homogenized for 15 s using a Polytron Homogenizer (Brinkman, Westbury, NY) and spun down at 20000 g for 30 min. Twenty μg protein from the resulting supernatant was applied in each well and separated by SDS-PAGE using NuPage pre-cast gels (Invitrogen, Carlsbad, CA). After transfer to a nitrocellulose membrane for 1 h at 30 V and subsequent treatment with blocking solution (TBS with 0.1% Tween and 5% milk powder) for 30 min at room temperature, the membrane was incubated overnight at 4°C in blocking solution containing anti-CD133 mouse monoclonal antibody (clone AC133 or AC141, Miltenyi) diluted 1:100. The primary antibodies were detected using a horseradish peroxidase-conjugated goat anti-rabbit/mouse secondary antibody diluted 1:20,000 (Immunotech, Fullerton, CA). The Western blot was developed using Supersignal West Pico Chemiluminescent Substrate (Pierce Biotechnology, Rockford, IL) and detected with a Fuji LAS 3000 Imager (Fuji Photo Film Co, Tokyo, Japan).

### Statistics

The statistical analyses were performed using the software package Statistica 4.1 (StatSoft Inc., Tulsa, OK). The Product-limit (Kaplan-Meier) Analysis Module was used for comparing survival between multiple groups. Survival times versus cumulative proportion surviving, according to breakdown by staining intensity groups 0, 1 and 2, were plotted.

## Results

### Specificity of CD133 staining

**Figure 1:**
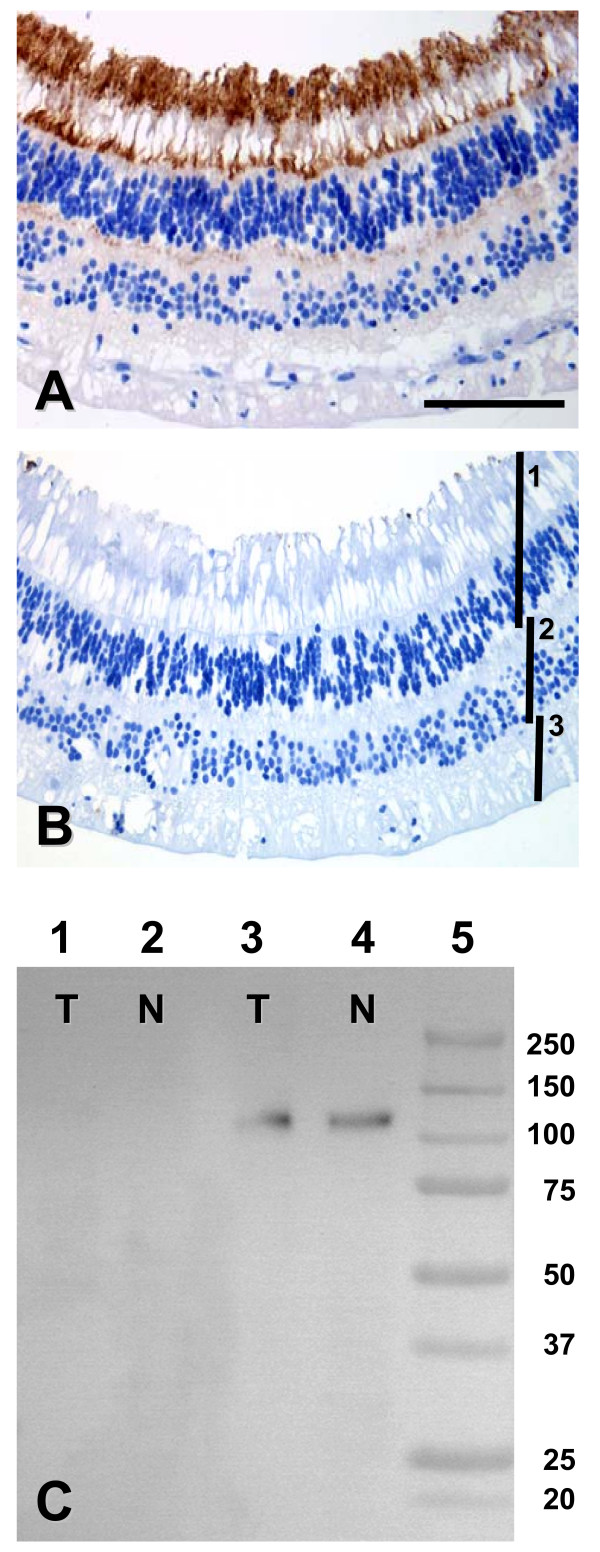
Control of the specificity of CD133 staining. **A) **A section of retina showing strong membranous and cytoplasmic staining concentrated in the apical part of both cone and rod cells. Weak positivity is noted in a layer basal to the cone and rod nuclei. **B) **A parallel retina section processed similarly to A, except from the addition of primary antibody. The cell layers are rods and cones (1), horizontal, bipolar and amacrine cells (2) and ganglion cells (3). A, B = ×400, scale bar = 100 μm. **C) **Western blot stained with the two antibodies AC141 (lanes 1 and 2) and AC133 (lanes 3 and 4). Only AC133 recognized the expected band of around 117 kDa. T and N denote protein extracts from a pancreatic ductal adenocarcinoma and from normal pancreas, respectively. The band sizes (kDa) of the molecular weight standard in lane 5 are listed to the right.

We tested three different antibodies against CD133 (Table 1) on formalin-fixed, paraffin-embedded human retina, a tissue with known CD133 expression [40]. The monoclonal antibody AC133 gave the most sensitive results with the lowest background staining (Figure 1, see also Materials and Methods). There was abundant cytoplasmic and membrane positivity in the apical part of the rod and cone cells and less intense staining basal to their nuclei, possibly representing the apical end of neighboring cells. Staining with the polyclonal antibody K-18 was weaker and more difficult to separate from the background, whereas the monoclonal antibody AC141, recognizing a different CD133 epitope than AC133, did not show a positive reaction on retinal tissue (not shown). The specificity of the AC133 antibody was confirmed by Western blotting of protein-extracts from normal pancreas and pancreatic ductal adenocarcinoma (Figure 1C).

### CD133 expression in normal and malignant tissues

**Figure 2:**
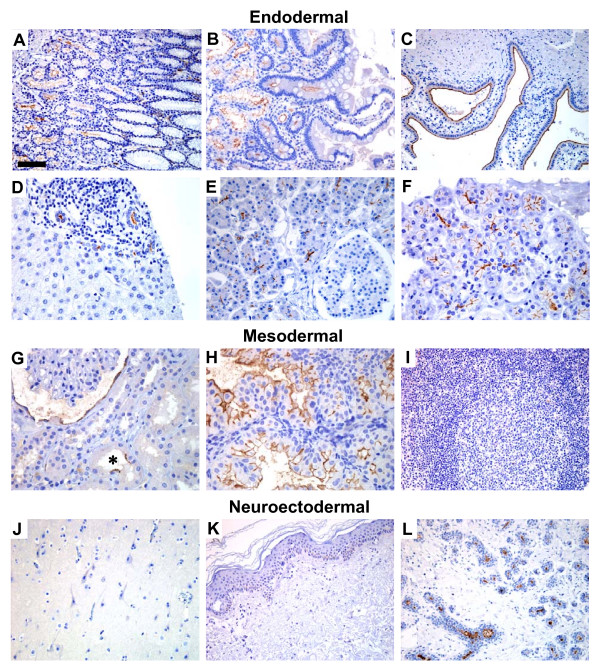
CD133 expression in endodermal, mesodermal and neuroectodermal tissues. Endodermal: **A) **stomach, **B) **duodenum, **C) **gall bladder, **D) **liver, **E) **pancreas, **F) **salivary gland. Mesodermal: **G) **kidney, **H) **endometrium in secretory state, **I) **lymphatic tissue (tonsil) with a germinal center. Neuroectodermal: **J) **brain (cortex cerebri), **K) **skin, **L) **mammary gland. Retina staining (neuroectodermal) is shown in Figure 1A and colon staining (endodermal) in Figure 3A. Note the apical/endoluminal membrane staining in glandular epithelia in general. In the stomach (A), duodenum (B) and colon (3A), CD133 expression is restricted to cells at the basal part of the mucous membrane. In the gall bladder (C), biliary ducts of the liver (D), endometrium (H) and mammary gland (L), there is homogenous apical membrane staining. In the pancreas (E) and the salivary gland (F), homogenous endoluminal staining of the smallest ductal structures is present. In the kidney (G), all cells of the Bowman's capsule seem to express CD133 in the cytoplasm and membrane, whereas apical membrane staining is present in some of the duct cells (asterisk). No staining was detected in tonsils (I), lymph nodes (not shown), cortex cerebri (J) or skin (K). Weak color in the cytoplasm of basal cells in the skin is supranuclear melanin pigment. A-C, I-L = ×200, scale bar in A = 100 μm. D-H = ×400, scale bar in A = 50 μm.

**Figure 3:**
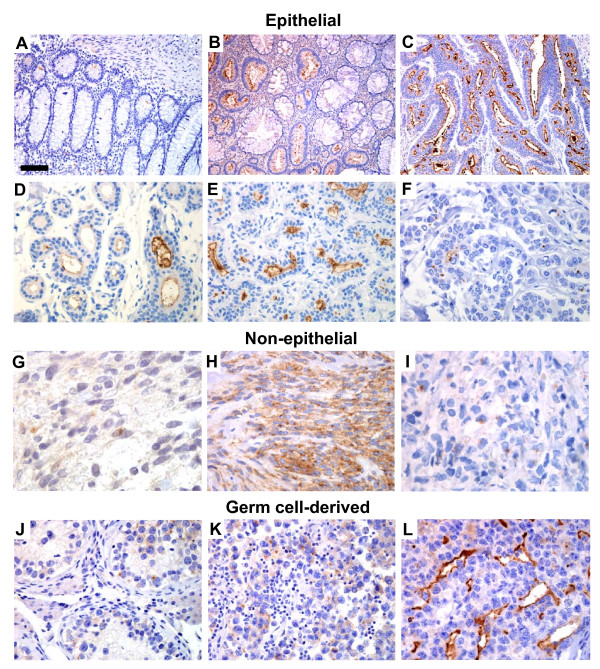
CD133 expression in normal and neoplastic tissues. Epithelial tissues and tumors: **A) **normal colon mucosa, **B) **colon adenoma, **C) **colon adenocarcinoma, **D) **normal mammary gland, **E) **mammary adenoma, **F) **mammary carcinoma. Non-epithelial malignant tissues: **G) **brain glioblastoma multiforme, **H) **gastrointestinal stromal tumor, **I) **myelogenous leukemia soft tissue infiltrate. Germ cell neoplasias of the testis: **J) **Intratubular germ cell neoplasia, **K) **seminoma, **L) **embryonal carcinoma. Note the apical/endoluminal membrane staining in epithelial tissues (A, D) and in neoplasias with tubule-forming tumor cells (B, C, E, F, L). In solid tumors without tubular structures there is perinuclear or diffuse cytoplasmic staining (G-K). A = ×200, scale bar = 100 μm. B, C = ×100, scale bar in A = 200 μm. D-F, H, J-L = ×400, scale bar in A = 50 μm. G, I = ×1000, scale bar in A = 20 μm.

We examined CD133 expression in endodermal, mesodermal and neuroectodermal organs of adult humans. In non-squamous, glandular epithelia, we observed an apical membrane staining of cells arranged in ductal structures (Figure 2). Squamous epithelia of the skin, adult non-epithelial tissues (fatty tissue, fibrous tissue, blood and lymphatic vessels, lymph nodes) and neural tissues (brain and peripheral nerves) were all negative (Figure 2 and not shown). In the stomach, duodenum and colon (Figure 2A, 2B, 3A), cells at the basis of the crypts – where stem cells are likely to reside – expressed CD133 at their apical/endoluminal surface. The staining pattern of the epithelia may reflect a particular stage in maturation as the more differentiated cell populations in the upper part of the mucous membranes were negative. In tubularly arranged epithelia of the liver, pancreas, salivary and mammary gland, CD133 expression started at the proximal end of the lumen and was maintained with increasing diameter of the duct (Figure 2D–2F, 2L).

We also stained tissues from a set of human malignancies: colon carcinoma, carcinoma of the mammary gland, glioblastoma multiforme (GBM), chronic myelogenous leukemia, gastrointestinal stromal tumor (GIST) and three types of testicular tumors (Figure 3). In malignant tissues, we observed two distinct staining patterns: There was apical/endoluminal membrane staining, opposite to the basal membrane, in the malignant glandular epithelia, whereas the non-epithelial tumors mainly exhibited perinuclear/cytoplasmic positivity. Moreover, in the infiltrating ductal carcinoma of the mammary gland (Figure 3F), distinct cytoplasmic globules expressing CD133 were seen where cytoplasmic lumina had been formed or where mucus had been retained in the cytoplasm.

In the normal tissues studied in Figure 2 as well as in the epithelial tumors of Figure 3, cytoplasmic CD133 staining was observed only in some rare, single cells. This contrasts to the staining pattern of the non-epithelial tumors (GBM, GIST, chronic myelogenous leukemia, intratubular germ cell neoplasia and seminoma) shown in Figure 3. Cytoplasmic CD133 expression appeared most abundant in the GIST (Figure 3H), and a series of 27 such tumors were examined, 22 (81%) being positive. There was varying CD133 intensity, not related to morphologic variations (not shown). In GBM and chronic myelogenous leukemia, only a few cells with perinuclear cytoplasmic staining were noted (Figure 3G, 3I). The same cytoplasmic staining but in more cells, was seen in intratubular germ cell neoplasia and seminoma (Figure 3J, 3K). The embryonal carcinoma (Figure 3L) did show strong endoluminal cell membrane staining whenever a lumen was seen, and also weak cytoplasmic staining.

The overall impression from the normal and malignant tissues depicted in Figures 2 and 3 is that CD133 positivity is related to tissue architecture. The protein is expressed apically in the membrane of epithelial cells when a lumen has been formed and in the cytoplasm of solidly arranged malignant tissues of non-epithelial origin. Staining was not restricted to organs arising from a particular embryonic layer.

### Expression in normal pancreatic tissue

**Figure 4:**
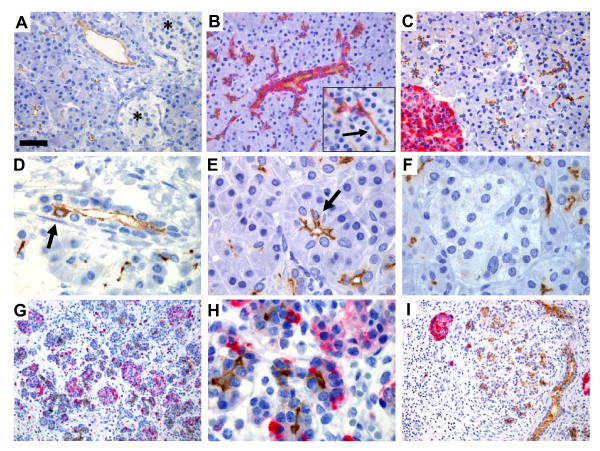
CD133 expression in adult and fetal pancreas. **A-F) **Normal adult pancreas. Note the apical/endoluminal staining of the ducts and that the islets of Langerhans (asterisks in A) are negative. See also Figure 2E. **B) **Double staining with CD133 (brown) and cytokeratin 19 (red) showing abundant co-expression of the two markers. The insert is a higher magnification showing CD133-positive, CK19-negative cells in the region connecting ducts and acini (arrow). **C) **Double staining with CD133 (brown) and chromogranin A (red). No co-expression was detected. **D) **A duct with a single cell exhibiting cytoplasmic CD133 positivity (arrow). **E) **Acini with apical/endoluminal expression whenever a visible lumen is present. The arrow points to a cell in centroacinar position, apparently having cytoplasmic expression. **F) **A CD133-negative islet. **G, H) **Fetal pancreas at gestational week 18, low and high magnification. Staining as in C. **I) **Chronic inflammation and atrophic exocrine tissue. Staining as in C. Note the similarity to G in general architecture and the difference in distribution of chromogranin A-positive endocrine cells. A-C = ×400, scale bar in A = 50 μm. D-F, H = ×1000, scale bar in A = 20 μm. G, I = ×200, scale bar in A = 100 μm.

We then focused our study on pancreatic CD133 expression. In normal pancreas, CD133-positive cells were seen in the center of the acini whenever a small lumen was present (Figure 4). The positivity continued in the following ducts and, with variable and decreasing intensity, into the larger ducts. Cells expressing CD133 on their apical/endoluminal surface showed wide co-expression of CK19 in their cytoplasm. CK19 staining apparently followed a gradient with strongest intensity in the larger ducts, fading towards the periphery and negative in the tiniest ducts or centroacinar cells which still showed some CD133 expression (Figure 4B). No co-expression of CD133 and chromogranin A was observed (Figure 4C). Although staining was seen at the apical membrane in the vast majority of cells, single cells in some ducts showed strong cytoplasmic staining (Figure 4D). In the acini (Figure 4E), a few cells located near the luminal surface showed strong CD133 expression suggesting cytoplasmic staining in the centroacinar cells, but these are difficult to separate from acinar cells by light microscopy only. Staining was never seen in the islets of Langerhans (Figure 4A, 4F). To verify that the epitope(s) recognized by the antibody clone AC133 were not influenced by the processes of formalin fixation, dehydration and antigen retrieval, we stained cryosections of normal human pancreas. The staining pattern was the same as described above, with apical/endoluminal CD133 positivity in the proximal ducts and completely negative islets (not shown).

In fetal pancreatic tissue (Figure 4G, 4H), the ductal cells and cells of the center of small acini showed CD133 positivity but the staining appeared less distinct than in tissue from adult pancreas. Tissue morphology and staining pattern were strikingly similar in normal fetal pancreas and pancreatic tissue with fibrosis and atrophy of the exocrine tissue in adults (Figure 4I). In fetal tissue the chromogranin A-positive endocrine cells were intermingled between acinar and duct cells and the endocrine and exocrine cells were not as clearly separated as in the adult pancreas (Figure 4G, 4H). In the normal pancreas, the antibody K-18 (Table 1) stained the same cells as AC133, but more weakly (results not shown).

### Expression in pancreatic ductal adenocarcinomas

**Figure 5:**
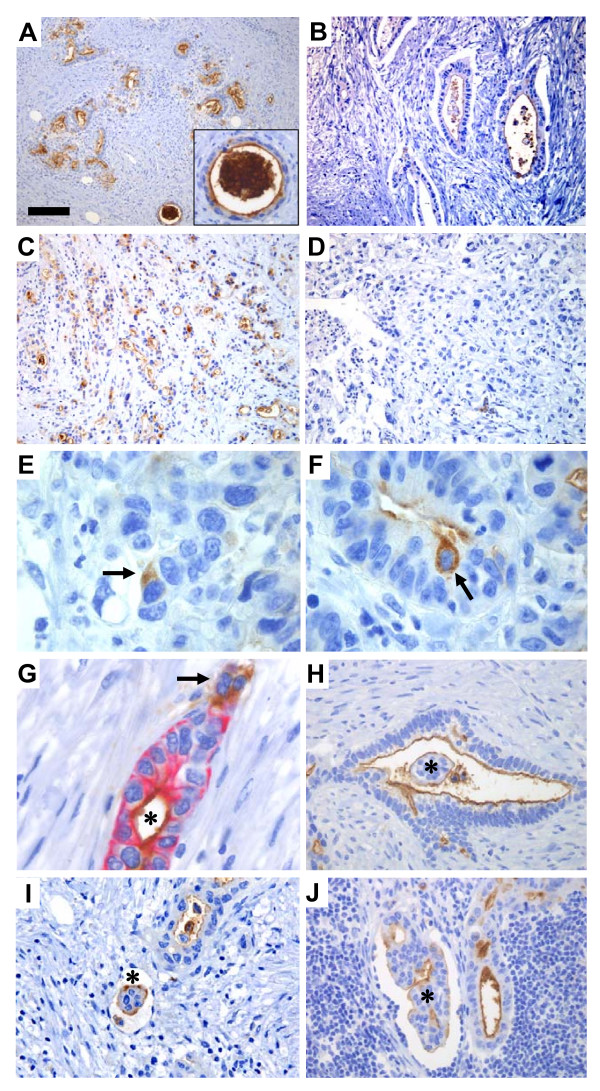
CD133 expression in pancreatic ductal adenocarcinomas. **A-D) **Variable expression in carcinomas of high (A, B) and low (C, D) differentiation grade. The insert in A is a high magnification of a cancerous duct with intense CD133 positivity in amorphous, intraductal material. **E, F) **Rare, single tumor cells with cytoplasmic staining (arrows). **G) **CK19-CD133 double staining of a malignant duct. Note cytoplasmic CD133 expression (brown) in a CK19-negative cell group (arrow) and co-expression of both markers in cells surrounding the lumen (asterisk). **H-J) **Staining (asterisks) at the apical/endoluminal membrane surface and surface of papillary arranged tumor cell clusters in a malignant duct (H), in a vessel of a primary tumor (I) and in a vessel of a lymph node metastasis (J). A-D = ×200, scale bar in A = 100 μm. E-G = ×1000, scale bar in A = 20 μm. H, J = ×400, scale bar in A = 50 μm. I = ×630, scale bar in A = 32 μm.

**Figure 6:**
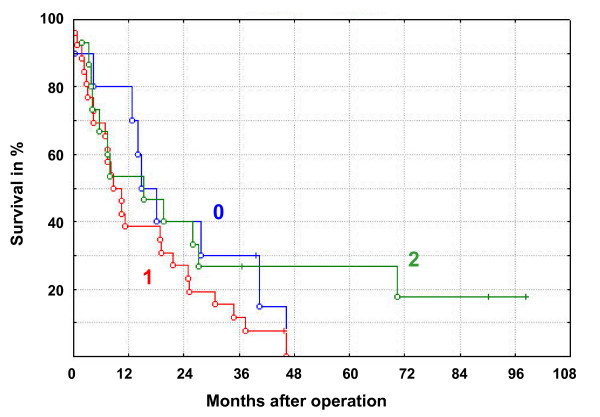
Cumulative proportion survival (Kaplan-Meier) plot for 51 pancreatic adenocarcinomas according to breakdown by CD133 expression (negative = 0, weakly positive = 1, strongly positive = 2). Median survival time was 16, 9 and 15 months for groups 0, 1 and 2, respectively. The observed survival times are indicated by circles (complete) or crosses (censored observations).

CD133 expression was evaluated in a series of 51 pancreatic ductal adenocarcinomas in TMA blocks. Forty-one cases (80%) were positive, and expression was mainly seen in the apical/endoluminal cell surface in malignant ductal structures (Figure 5A–D). In the positive cases, expression area and intensity varied along with the morphological heterogeneity of the tumor. The CD133 expression pattern in colon and mammary gland adenocarcinomas could indicate higher expression in lower grade tumors. This was not true for pancreatic adenocarcinomas in our series. Some well-differentiated/low-grade tumors with mucous production had low CD133 expression (Figure 5B) and some low-differentiated/high-grade tumors still formed tubuli with abundant endoluminal CD133 expression (Figure 5C). Moreover, there was no significant correlation of CD133 expression with survival, TNM tumor stage or differentiation grade in our series (Figure 6 and not shown). Interestingly, debris in small and dilated ducts lined by malignant epithelium was strongly stained in positive cases (Figure 5A) and also found in cases otherwise weakly positive (Figure 5B).

Cytoplasmic CD133 staining was seen in less than 1% of the malignant epithelial cells (Figure 5E, 5F) as estimated from the ten cases where a whole section of tumor tissue was available for examination. In a well-differentiated pancreatic cancer double-stained with CK19 (Figure 5G), we could demonstrate a similar staining pattern as seen in the normal pancreas in areas where acini and ducts connect: Cytoplasmic CK19 was co-expressed with membranous CD133 in apical/endoluminal cells lining the ductal lumen whereas some cells expressed cytoplasmic CD133, but not CK19, at the site of solid growth.

In papillary arranged tumor cell groups that did not have a central lumen, CD133 staining was turned "inside-out" (Figure 5H). This phenomenon was seen especially in cancer cell groups inside vessels (Figure 5I) and in addition on the entire surface of shed single cells, lying in malignant ducts (Figure 5B, 5H). In investigated lymph node metastases, malignant cells did mirror the focal, apical/endoluminal membrane staining of CD133 in the primary tumors (Figure 5J). The phenomenon of outer surface staining of tumor cells infiltrating vessels was also observed in lymph nodes (Figure 5J).

## Discussion

We have examined CD133 expression in normal and malignant human tissues with special emphasis on pancreas and pancreatic ductal adenocarcinomas. In the normal tissues, expression was seen mainly at the apical/endoluminal surface of non-squamous, glandular epithelia and could not be related to a specific embryonic layer of organ origin. A similar staining pattern was seen in malignant epithelial tissues. However, in some non-epithelial malignancies cytoplasmic positivity was observed either diffusely in the cytoplasm of the majority of tumor cells (GISTs) or as discrete, perinuclear dots in a few tumor cells (GBM, myelogenous leukemia). Such perinuclear CD133 staining in a few cells have also been reported in the sub-ependymal zone of fetal human brain [48]. In germinal cell-derived tumors, staining varied according to the architecture. In summary, CD133 was located at the apical/endoluminal surface of cells forming lumina and in the cytoplasm of cells when they exhibited solid growth.

### Pancreatic expression of CD133

In the normal pancreas, CD133 is expressed at the apical/endoluminal surface of ductal cells. The expression appeared more pronounced towards the acini than towards the larger ducts. CD133 and CK19 (a marker for ductal cells) were generally co-expressed in the fully differentiated ductal epithelium. CD133-positive and CK19-negative cells were present in the region connecting the smallest ducts and secretory acinar cells. In this region there were some single cells in a centroacinar position with mainly cytoplasmic CD133-staining. Cytoplasmic expression was also observed in single ductal cells. They did not differ in their morphology or position from neighboring cells. We therefore conclude that they do not correspond to the so-called "helle Zellen" [49], members of the diffuse neuroendocrine system of the pancreas.

Areas where epithelia of divergent differentiation coincide have been proposed as locations (niches) for organ-specific stem cells in intestine and liver [50-52]. Recently the centroacinar cells of the pancreas have come into focus as a probable cell of origin in pancreatic intraepithelial neoplasia (PanIN), thereby also becoming candidates for the progenitors of ductal adenocarcinomas [43]. We note that the centroacinar cells may appear to be demarcated by cytoplasmic CD133 staining and CK19 negativity. It is therefore tempting to speculate that these cells form a subpopulation with a specific role in pancreatic exocrine tissue.

Both in the normal pancreas and in the ductal adenocarcinomas, CD133 is expressed in far too many cells to be a specific (cancer) stem cell marker. Notably, a high percentage of CD133-expressing cells (up to 24%) was reported also in colon cancer [26], but there were no data about subcellular localization. We suggest that CD133-expressing cells in the pancreas, as demonstrated by the AC133 antibody, include at least two subpopulations. The main population expresses CD133 at the cell surface and represents a particular stage in cell differentiation connected to the formation of lumina and ducts. The minor population has mainly cytoplasmic staining and represents less than 1% of epithelial cells, both in normal pancreas and in the ductal adenocarcinomas. If the population with cytoplasmic CD133 expression could be shown to serve a role as stem cells in the normal tissue, it would become an interesting candidate for the transformation to tumor-initiating cells in pancreatic cancer.

### CD133 as a potential clinical marker in pancreatic cancer

Pancreatic ductal adenocarcinoma is usually detected at an advanced stage and is one of the human cancers with the worst prognosis. Earlier detection will probably be essential for improving patient survival. Biochemical or molecular analysis of pancreatic juice could be one option for increasing the likelihood of early diagnosis. Yoshida et al. [41] investigated purified ductal cells from the pancreatic juice of healthy individuals and cancer patients, and demonstrated upregulation of CD133 mRNA in some cases. We here show expression of CD133 protein in the epithelium lining the ducts of both normal and cancerous exocrine tissue. We also observed shed, CD133-expressing tumor cells and apparently non-cellular, CD133-positive material in the malignant ducts. Thus, a quantitative analysis of CD133 in pancreatic juice from suspected cancer patients might be of interest, in particular with regard to whether it could aid the discrimination between chronic inflammation and malignancy.

Intriguingly, we noted CD133 expression on the outer surface of tumor cell groups in small lymphatic or blood vessels. CD133 has been detected on extra-cellular membrane particles isolated from neuroepithelial and epithelial cells in various body fluids [53]. Moreover, in a series of patients suffering from colon cancer, Lin et al. [54] discovered that a high level of CD133 mRNA in blood predicted disease recurrence. Their interpretation was that bone marrow-derived, endothelial progenitor cells and not cancer stem cells were the source of the elevated CD133 mRNA levels. Nevertheless, CD133 mRNA and/or protein levels in blood could be of predictive or even diagnostic value. Thus, the results of Marzesco et al. [53] and Lin et al. [54] along with our own observations warrant further studies of CD133 mRNA and/or protein in blood samples from pancreatic cancer patients.

### CD133 expression and implications for cell sorting

According to the literature, the distribution of CD133 mRNA and results from immunohistochemistry using AC133 as primary antibody is not completely concordant [38]. Moreover, many tissues where we observed positive staining have previously been reported negative for AC133 by immunohistochemistry [33,38]. There are many explanations for the observed discrepancies. Our protocol of immunostaining on formalin-fixed, paraffin-embedded tissue involves a relatively high concentration of the AC133 antibody and a sensitive detection system, indicating that there may be problems in epitope retrieval or antibody sensitivity in fixed material. In addition, the antibody clone AC133 recognizes an epitope containing a potential glycosylation site [37], which suggests that the non-glycosylated form may escape detection. There are also a multitude of CD133 mRNA splice variants [55] and neither AC133 nor other antibodies can be expected to cover all protein variants that may be produced by the different mRNAs.

Several seminal studies [5,6,26,27,31,56,57] have used AC133 as a tool in fluorescent-activated cell sorting (FACS), isolating normal or malignant cells with stem cell-like properties. FACS requires tissue dissociation. The mechanical and enzymatic procedures necessary to make single-cell suspensions disrupt the cellular microenvironment and can be expected to alter the expression of both extra- and intracellular proteins, thereby influencing the selection of the cell population to be used in subsequent transplantation experiments. Despite the central role of CD133 as a marker for selecting postulated cancer stem cells, it is not known how this protein is affected by the procedures used for tissue dissociation and this needs to be addressed experimentally.

The data acquired by CD133-positive cells sorted from single-cell suspensions derived from solid tissues must therefore be interpreted with caution, especially since the physiological function of the CD133 protein may be connected to cell polarity [58] and to cell orientation in tissues [35]. Such a role would be in accordance with the apical/endoluminal staining pattern that we report in this paper. How the cell populations that we detect by immunohistochemistry on formalin-fixed, paraffin-embedded tissue relate to those sorted by FACS using the same primary antibody, remains to be studied. One possibility is that the cells with abundant cytoplasmic expression are those with the highest level of CD133 protein in the membrane.

### The function of CD133

The physiological role of CD133 is elusive. As discussed above, studies are complicated by the occurrence of many mRNA splice variants and possibly also by a changing glycosylation status of the protein. There are several commercially available antibodies towards human CD133, yielding overlapping but different staining patterns (own unpublished data). Moreover, the protein is present at different locations in the cell (cytoplasm or membrane), which in turn may reflect specific cellular functions. Taken together, our data on CD133 expression in the pancreas as well as in other organs indicate that the protein relates to tissue architecture, apical/endoluminal membranous staining of CD133 being a characteristic of well-oriented, polarized and differentiated cells of glandular epithelia.

As mentioned, CD133 has been implicated in cell polarity, which is required for cell movement [58,59]. The latter is crucial for processes such as chemotaxis, embryonic development, invasive growth and metastasis, but not in itself regarded a stem cell property. However, cell polarity is also important for asymmetric cell division, an inevitable characteristic of stem cells [60]. Kosodo et al. [61] described the asymmetric distribution of a minute fragment of the apical plasma membrane expressing CD133 to the daughter cells in asymmetric neurogenic divisions during central nervous system development. Dubreuil et al. [62] showed CD133 expression in apical midbodies during symmetric cell divisions of neuroepithelial cells and the release of these apical midbodies in the neural tube fluid. Both studies link the plasma membrane domain in which CD133 is included, to the type of cell division. The existence of CD133-positive, non-cellular and therefore probably plasma membrane fragments in our series of pancreatic ductal adenocarcinomas could be a result of CD133 release during proliferative symmetric cell divisions, the main type of cell divisions responsible for tumor growth. It is a further argument for investigating pancreatic juice and blood of pancreatic cancer patients for CD133 at the protein level.

A stem cell population is characterized by the lack of differentiation markers and the ability to undergo multi-lineage differentiation. For the cancer stem cell hypothesis, however, xenograft tumor models have been an essential experimental approach. In several such studies, CD133 was hailed as a cancer stem cell marker [6,22-28]. It is, however, doubtful that CD133 expression in tumor-initiating cells is an absolute necessity in xenograft models, as it recently was shown that human brain tumors can be transplanted from CD133-negative cancer cells [63,64]. Results from xenograft experimental models have therefore to be carefully interpreted [48]. Tumor initiation at other places than the primary organ of origin and in a different species is probably a fine-tuned process where the microenvironment and immunity is of major importance. It will be essential to uncover the physiological role of CD133 for determining whether it is a marker for a true cancer stem cell population or a marker for cells with enhanced capability to proliferate in other species [65].

## Conclusion

CD133 expression in the form of the epitope recognized by the antibody AC133 was not related to a specific embryonic layer of organ origin. The protein was found at the apical/endoluminal surface of glandular epithelia and of malignant cells in ductal arrangement. In pancreatic ductal adenocarcinomas, CD133 was present in shed tumor cells and on the surface of tumor cell groups in vessels, suggesting a potential as clinical marker. Neither in the pancreas nor in the other investigated organs can CD133 membrane expression alone be a criterion for "stemness".

## Competing interests

The author(s) declare that they have no competing interests.

## Authors' contributions

HI designed the study, did the evaluation of the histology and immunohistochemistry, and wrote the manuscript. DH performed the surgical operations, evaluated the clinical records and did the statistical analysis. PØS participated in the design of the study and performed the Western blot. OJS assisted in the method development and did the final immunohistochemistry. AM designed and lead the study, did the evaluation of immunohistochemistry, and wrote the manuscript. All authors have read and approved the final manuscript.

## Pre-publication history

The pre-publication history for this paper can be accessed here:


